# Prostate Adenocarcinoma Within a Thymoma: A Rare Case of Tumor-to-Tumor Metastasis

**DOI:** 10.7759/cureus.33537

**Published:** 2023-01-09

**Authors:** Simone Furia, Lorenzo Nicole', Licia Laurino, Cristiano Breda

**Affiliations:** 1 Thoracic Surgery, Ospedale dell'Angelo, Venezia-Mestre, ITA; 2 Pathology, Ospedale dell'Angelo, Venezia-Mestre, ITA; 3 Department of Medicine, University of Padova, Padova, ITA

**Keywords:** malignant thymoma, prostatic adenocarcinoma, thymic tumors, thymus, tumor-to-tumor metastasis

## Abstract

Tumor-to-tumor metastasis is defined as when metastasis from a primary tumor (donor) grows in a different primary neoplasm (recipient). Due to the structure of the thymus and the low incidence rate, thymic epithelial neoplasm has been rarely described in the literature as a recipient for metastases.In this report,a patient with advanced prostatic cancer and under control after chemo/hormone therapy was directed to our thoracic surgery unit for an anterior mediastinal mass detected during the staging workup for prostate disease. A limited uptake at fluorodeoxyglucose-positron emission tomography (FDG-PET) in the mediastinal lesion, while the surrounding tissue showed diffusely negative hypermetabolism, suggested a second primary thymic epithelial tumor with a possible carcinomatous differentiation. A thymectomy through a median sternotomy was carried out. Histopathological analysis after thymectomy revealed a type A thymoma with multiple elements of prostate adenocarcinoma within it. The foci of prostate adenocarcinoma were co-located in the context of the thymoma, revealing what is defined as a tumor-to-tumor metastasis.To our knowledge, this is the first report describing a thymoma as the recipient of metastases coming from a primary extrathoracic tumor without the involvement of other thoracic organs.

## Introduction

Tumor-to-tumor metastasis (TTM) is a rare phenomenon, and defined by the criteria proposed by Campbell [1] as when the evidence of a metastasis in the host tumor is the result of a true pathologic spread and not a consequence of contiguous growth (collision tumor). To date, more than 100 TTM cases were published in the literature and lung cancers are the most common donors in TTM, followed by carcinomas of the breast, gastrointestinal tract, prostate, and thyroid. The most frequent recipient tumor is renal cell carcinoma, followed by meningioma and thyroid tumor [2-5]. Thymoma is considered a rare neoplasm with an overall incidence of 1.5 cases per million whereas the incidence of thymic malignancies is 0.13 cases/100.000 persons/year [6]. In the context of a thymoma, the onset of metastases from an extrathoracic primary tumor is an unexpected event that could represent a diagnostic challenge. In our report, a patient with advanced prostatic cancer with multiple bone metastases underwent sternotomy for an anterior mediastinal mass, suspected of a concomitant primary thymic epithelial tumor. Pathologic report after thymectomy documented the simultaneous presence of a type A thymoma and a specimen positive to immunochemical markers for prostate adenocarcinoma, consistent with the criteria proposed for the diagnosis of tumor-to-tumor metastasis.

## Case presentation

**Figure 1:**
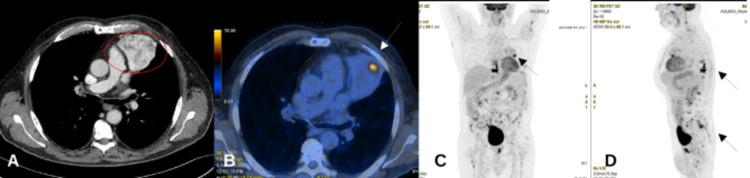
Imaging of the mediastinal neoplasm at CT scan and at FDG-PET scan The mediastinal mass of 81x73x54mm (red circle) at CT-scan (A). FDG-PET scan: a focal hypermetabolism with SUVmax 11.30 (white arrow) within negative surrounding tissue (B). Advanced prostatic adenocarcinoma with multiple metastases at the thoracic vertebrae, femur, sacrum and within the mediastinal lesion (black arrows) (C, D).

We present the case of a 75-year-old man, a current smoker (10 pack/year) with a history of arterial hypertension and gout with arthropathy. For the onset of nocturnal polyuria, the PSA level was obtained, resulting in a value of 300 ng/ml when the normal age-adjusted serum-PSA reference range is 0.0-6.5 ng/mL. For the diagnostic work-up, an abdomen CT scan and a trans-rectal ultrasound-guided biopsy (TRUSB) were carried out. At the imaging, a prostate increased in volume and multiple osteoblastic bone lesions, localized in the sacrum, femur, and in the spine were detected. A prostatic adenocarcinoma (Gleason 4+5), with infiltration into adipose tissue, was finally staged as cT4N2M1 (Stage IV). To complete the staging, a thoracic CT scan was required and a mass was discovered in the anterior mediastinum. The patient was initially treated with a combination of docetaxel for 6 cycles and androgen-deprivation therapy. After a good response to treatment, the PSA levels decreased to 5 ng/ml and alkaline phosphatase decreased from 269 U/L to 73 U/L (with a reference range of 30-120 U/L). Due to the decrease in prognostic markers for advanced prostate cancer after chemotherapy along with hormone therapy, the patient was referred to our Thoracic Surgery Unit for assessment of a mediastinal lesion, suspected to be a second primary tumor. An FDG-PET confirmed hypermetabolic spots in the femur, in the dorsal spine and showed an intense uptake enclosed in a limited area within the parasternal malignancy, suggesting a possible thymic carcinoma (Figure 1). A transthoracic needle biopsy was performed and the histological report documented a combination of epithelioid and spindle cells, strengthening the diagnostic hypothesis towards a thymic neoplasm rather than a prostate localization. After obtaining an agreement and involving our multidisciplinary team, the patient was admitted to our unit for thymectomy. After resection, the course was uneventful and the patient was discharged on the 6th postoperative day. At 2 months from surgery, chest radiograph findings were compatible with uncomplicated outcomes of a sternotomy. For the follow-up of his prostate cancer, the patient was referred to oncologists.

**Figure 2:**
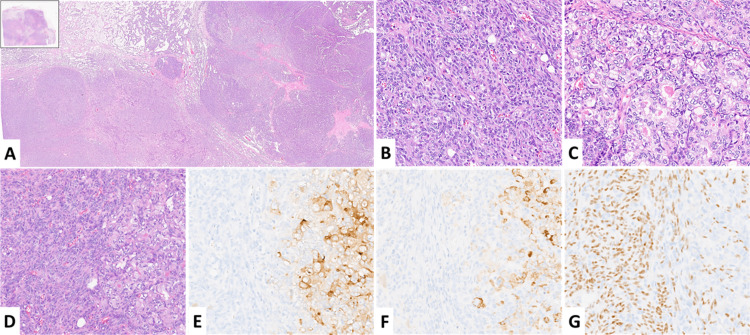
Pathologic specimen Panoramic overview at 100x magnification, showing multinodular growing pattern and the clear area corresponding to the prostatic carcinoma metastasis (right, A). Classical type A thymoma (B). Prostate cancer metastasis (C ). Detail showing metastatic tissue (right) with the thymoma component (left), (D). Immunostain of the area showed in D with PSA and racemase that highlight prostatic tissue (E and F, respectively), and with p63 that highlight thymoma. B,C,D,E,F original magnification 200x.

At gross pathological examination, the surgical specimen presented as an oval-shaped mass with a bosselated surface with a maximum diameter of 8.5 cm. The cut surface was lobulated, with focal cystic changes, light tan colored with scattered clear areas. No evidence of macroscopic infiltration of the capsula or of the mediastinal fat was observed. Microscopically, at low magnification, the tumor was surrounded by a complete fibrous capsule and displayed coarse lobulation with fibrous bands. Microcystic changes were confirmed in the subcapsular area. Clear areas were frankly evident within the tumor (Figure 2A). At high magnification, features of a classical type A thymoma were observed, which included a solid proliferation of fusiform cells with elongated nuclei, dispersed nuclear chromatin, and inconspicuous nucleoli. Neoplastic cells were arranged in a sheet-like and storiform growth pattern. The level of atypia was minimal and only scattered mitotic activity was noted (Figure 2B). Immunohistochemically, the tumor cells were positive for AE1-AE3 keratins and to p63 (Figure 2G). Few isolated TdT-positive immature T-cells were observed. Clear areas showed completely different histological features with atypical epithelioid cells, clear amphophilic cytoplasm, enlarged nuclei, and prominent nucleoli (Figure 2C, 2D). Tumor cells were arranged in solid sheets also forming gland structures. Altogether, these features were strongly suggestive of adenocarcinoma differentiation. Further immunohistochemical analyses revealed negativity in these cells for CD5 and CD117 (c-KIT), ruling out thymic carcinoma. Following the Masaoka-Koga classification system, this type A thymoma was staged as pT1aN0 (Stage I). According to the clinical history of the patients, prostatic markers such as PSA and racemase were tested and resulted in producing a strongly positive observation only within the clear areas, confirming the prostatic origin of the cells (Figure 2E, 2F).

## Discussion

For the pathogenesis of TTM, various mechanical and metabolic factors including cell signaling, loss of the tumor suppressor gene, and the high content of collagen and lipids can contribute to the creation of favorable conditions for organ-specific metastatic progression [7,8] as a result of the mutual dialogue between tumor cells (the seed) and the micro-environment of the distant tissue (the soil) [7-11]. The bloodstream and the vascular network can mechanically determine which organ will be crossed first, whereas the metabolic interactions between cancer cells and the target substrate are involved in the maintenance of growth. Theoretically, all organs can be a target substrate for metastasis; nevertheless, when compared to other tissues, a primary neoplasm should be the least conducive to the engraftment of other cells due to the hostile setting created by the competition for nutritional factors important for any rapidly growing tissue. For that reason, the onset of TTM is assumed to be ruled by incidental events rather than by a selective spread to a distant site. Three requirements of the recipient tumor should be present for TTM: 1) hypervascular network to allow for hematogenous diffusion 2) rich blood supply for adequate nourishment, and 3) slow growth to provide enough time for the TTM to develop [11].

The thymus is an uncommon site for metastases. Middleton found an incidence rate of 7% among 102 thymic malignancies in his historical series of autoptic cases [12]. This depends on the specific structure of the thymic parenchyma involved in the regulation of cellular immune-competence. The blood-thymus barrier and the efferent lymphatic vessels decrease the likelihood of a direct contact with antigens or cancer cells spreading through the bloodstream or lymphatic circulation, although this contact remains possible through the connective tissue of the interlobular thymic septa.

In our report, at staging CT scan for prostatic cancer, a solid thoracic formation with a diameter of 81 mm was revealed (Figure 1A). The localization in the anterior mediastinum with a non-homogeneous contrast enhancement suggested a thymic malignancy. However, the presence of an extrathoracic primary tumor represented a diagnostic challenge. The FDG-PET showed in a focal point with a higher uptake with a SUVmax 11.30, while in the surrounding tissue, the metabolic rate remained diffusely low, as predictable in a low-risk thymoma (Figure 1B-D). The role of FDG-PET in the diagnostic workup and management of thymic epithelial tumors has been considered controversial [13,14], nevertheless, in accordance with the WHO classification, recent studies found a correlation between SUVmax and the grade of malignant thymoma [14,15]. Outlining the prognostic value, it might be defined into two subgroups: low-risk thymoma (type A, AB, B1) and high-risk thymoma (type B2, B3), while the thymic carcinoma (type C) is defined as a separate subgroup with the poorest prognosis. Some researchers indicated the SUVmax≥6 as the cut-off for high-risk thymoma and carcinoma [15]. In our case, the likelihood of a carcinomatous differentiation pushed clinicians to recommend a thymectomy.

For the pathologic analysis, there are two main lessons that have been learned from this case. Firstly, careful examination of thymic tumors should always be performed, and heterogeneous tissue areas must be identified and sampled. Functional imaging consultation should also be considered, as suggested by this case, in which a limited area with increased glucose uptake was clearly observed within the tumoral mass by FDG-PET. Such findings should be considered suggestive of carcinomatous transformation and for legitimate extensive sampling. Secondly, every case should be assessed within its specific clinical context. Thymic carcinoma usually arises de novo, but in some instances, foci of carcinomatous transformation may be recognized within a thymoma [16]. In this case, the status of advanced prostatic carcinoma with multiple metastases played a pivotal role to solve the correct diagnosis, as it prompted us to test prostatic markers such as PSA and racemase which gave a positive result as shown in Figure 2. Negativity for CD5 and CD117, typically expressed in thymic carcinoma, was not enough to exclude this diagnosis as it is well established that both markers may be negative in poorly differentiated thymic carcinoma [17], making this diagnosis to be considered in absence of clinical data as well as an immunohistochemical panel or prostatic markers.

## Conclusions

This is the first report, to our knowledge, of a thymoma as a recipient of metastases from primary extrathoracic neoplasms. The other two reports in the English literature on TTM within a thymic epithelial malignancy described metastases arising from lung cancer and breast cancer, which are defined as the most common donor. TTM in patients with multiple tumors represents a challenge for clinicians and pathologists, which necessitates awareness to collect the most complete medical history of a patient and to identify the separate tumor components, even in tissues considered as a rare sites for metastases.
